# Instantaneous Flow Structures and Opportunities for Larval Settlement: Barnacle Larvae Swim to Settle

**DOI:** 10.1371/journal.pone.0158957

**Published:** 2016-07-27

**Authors:** Ann I. Larsson, Lena M. Granhag, Per R. Jonsson

**Affiliations:** Department of Marine Sciences, University of Gothenburg, Tjärnö, Strömstad, Sweden; University of Lincoln, UNITED KINGDOM

## Abstract

Water flow affects settlement of marine larvae on several scales. At the smallest scale local flow regime may control the probability of adhesion to the substrate. Our aim was to mechanistically understand the transition from suspended to attached larvae in turbulent flow. Recently it was proposed that opportunities for larval settlement in turbulent boundary layers depend on time windows with suitable instantaneous flow properties. In flume flow we characterized the proportion of suitable time windows in a series of flow velocities with focus on the near-bed flow. The change in the proportion of potential settling windows with increasing free-stream velocities was compared to the proportion of temporary attachment of barnacle cypris larvae at different flow velocities. We found large instantaneous flow variations in the near-bed flow where cyprid attachment took place. The probability of temporary attachment in cyprids declined with local flow speed and this response was compatible with a settling window lasting at least 0.1 s with a maximum local flow speed of 1.9–2.4 cm s^-1^. Cyprids swam against the near-bed flow (negative rheotaxis) and the swimming speed (1.8 cm s^-1^) was close to the critical speed that permitted temporary attachment. We conclude that temporary attachment in barnacle cyprids requires upstream swimming to maintain a fixed position relative to the substrate for at least 0.1 s. This behaviour may explain the ability of barnacles to recruit to high-flow environments and give cyprids flexibility in the pre-settlement choice of substrates based on flow regime.

## Introduction

In the marine environment, transfer of propagules from pelagic to benthic habitats is a crucial step in many life cycles. The rates at which spores or larvae encounter and adhere to a substrate may strongly affect settlement patterns [1–4] and will influence population dynamics in sessile or sedentary organisms [5–7]. Coastal circulation is important for the transport of propagules to potential settlement sites [8], and on larger scales settlement patterns of sessile marine invertebrates are often linked to larval supply [9–12]. However, independent of larval supply, variation in local hydrodynamics between adjacent bays or sites within bays have recently been found to generate differences in settlement rates [3,13,14]. Small-scale hydrodynamics at the bed affect larval settlement patterns both through passive and active processes. The delivery rate of larvae to the substrate is mainly a passive process governed by turbulent mixing [15,16] although behavioural control of larval vertical velocity may modify the probability of bed encounter [17–20]. After contact, flow-induced forces may prevent propagules from attaching to the substrate [21–22]. Active behavioural response to flow at the substrate may also affect final settlement depending on choice of micro-flow environments and through decisions to leave the surface returning into the water column [1,4,23,24].

Most marine propagules settle within a more or less developed turbulent boundary layer [25]. Even if the mean flow velocity in a turbulent boundary layer increases predictably with the distance from the substrate there are large and chaotic fluctuations in time. Depending on, e.g. bed topography, waves and wind the turbulence intensity may differ greatly for a given mean flow velocity. Larvae or spores approaching the substrate interact with the instantaneous local flow and an improved mechanistic understanding of the settling process requires detailed information about the distribution of flow velocities on temporal and spatial scales relevant to contact, adhesion and behavioural responses [26–28]. Turbulent flow near the substrate is very dynamic, and high-velocity vortices known as sweeps will reach all the way down to a fraction of a millimetre away from the substrate with velocities in the same order as the mean free-stream velocity [29,30]. Consequently, the near-bed flow is expected to vary in time and space, potentially affecting spore and larval settlement. Crimaldi et al. [26] first suggested that larvae making contact with a substrate need some minimum time interval below a critical local stress to allow for attachment. The frequency of these so called settling windows in a particular flow regime will then determine the probability of successful larval attachment, a concept that has been further explored by Reidenbach et al. [27] and Koehl et al. [28] for various habitats and flow conditions. Possible mechanisms defining the settling window are the flow-induced forces at adhesion and the time required to make a secure attachment [15,31]. A larva or spore attaching to the bed is subjected to drag and lift forces induced by the flow. To attach and remain attached the adhesion strength of the propagule needs to resist the induced hydrodynamic forces [15].

**Fig 1:**
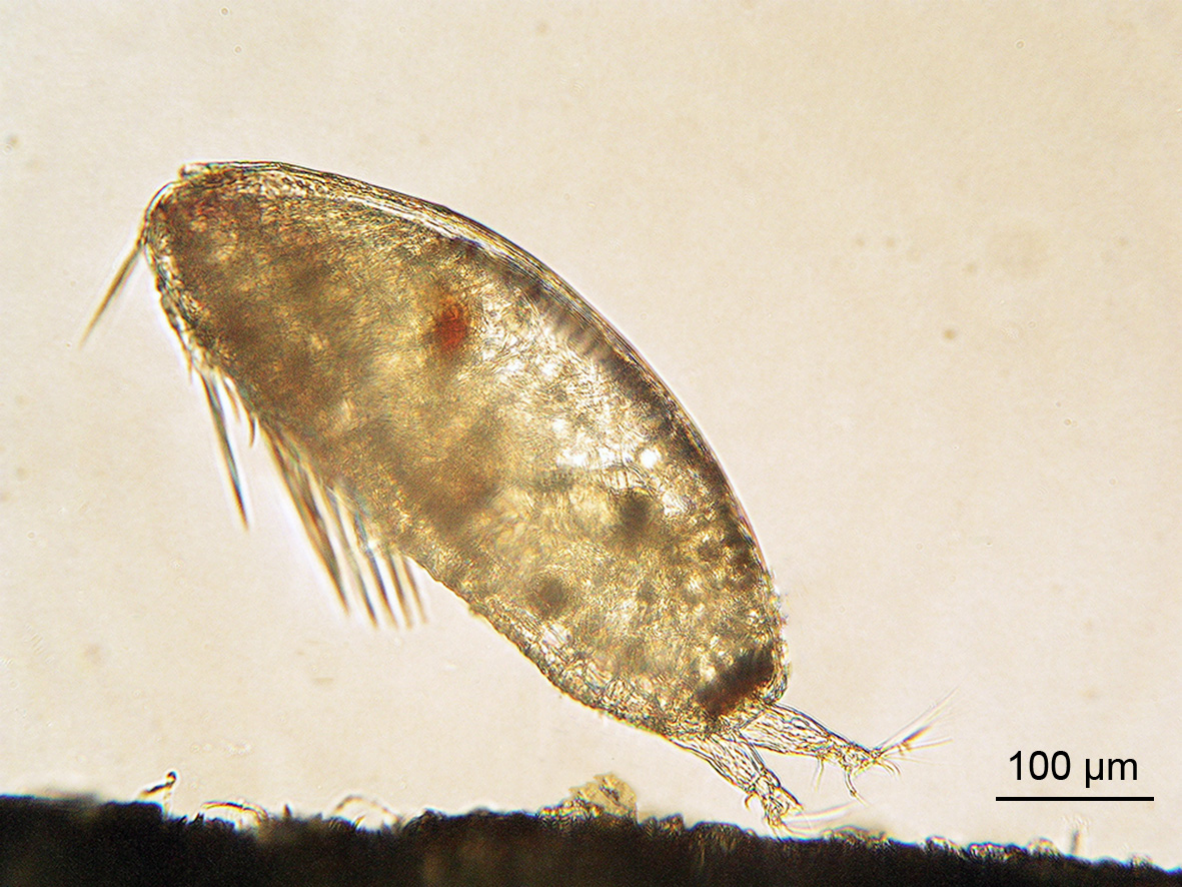
Cyprid of *Balanus improvisus* barnacle. The cyprid is the settling stage of the barnacle here making contact with the substrate before temporarily attaching with its antennular discs. Photo credit Kent Berntsson.

Cypris-larvae of rocky-bottom intertidal barnacles make a strong temporary attachment with the antennular discs [32,33] (Fig 1). This temporary attachment is achieved by a combination of cuticular villi covering the antennular disc and secretion of a viscoelastic adhesive [34]. In temporary attachment the cyprid may walk with its antennules to explore the substrate for suitable settlement sites, or the cyprid may decide to leave an undesirable substrate [35,36]. It is, however, unclear how the ability for temporary attachment is controlled by flow in the boundary layer [33,37]. In a field experiment, cyprids of *Balanus (Amphibalanus) improvisus* Darwin 1854 showed reduced settlement and recruitment with increasing local flow speed although modelled larval contact rate increased with flow velocity [3]. The negative correlation between local flow speed and recruitment suggested a regulating mechanism operating immediately after initial contact but before temporary attachment. The potential to remain in contact with a substrate in flow includes both passive and active mechanisms: 1) adhesion to surfaces, e.g. hydrophobic interactions [38], 2) gravity may prevent propagules from becoming resuspended on horizontal surfaces, 3) mechanical interactions with a substrate using protruding spines and setae [15], 4) larvae may behave actively to make contact or maintain a position at the substrate through directional swimming. To go from contact to temporary attachment a cyprid must orient the body to a position that makes temporary attachment with the antennular discs possible, and probably some time interval of low flow velocity relative the substrate is required. Crisp noted that barnacle cyprids swam upstream when close to the substrate (negative rheotaxis) [37]. He hypothesized that this was an adaptation to reduce the relative velocity to the substrate to allow temporary attachment in flow.

Previous studies of expected larval settling probability as a function of the frequency of settling windows have been performed under various flow conditions [26–28]. Reidenbach et al. [27] used values for larval adhesive strength to predict settlement probabilities of nudibranch larvae on coral reefs. However, empirical data of how settling windows correspond to actual larval attachment have never been collected. In this study we specifically studied how temporary attachment in barnacle larvae is attained and how attachment is controlled by boundary layer flow conditions. We first characterized the turbulent flow structures in the near-bed layer at the height of settling larvae. We did this by estimating the proportion of time in lull periods as a function of both critical bed shear stress and critical local velocity allowing temporary attachment. We also calculated the hydrodynamic forces imposed on cyprids at the bed and compared those to previously reported data of forces required to dislodge cyprids. The proportion of time for flow events acting as settlement windows and calculations of induced forces were then compared to empirical data on attachment probability and swimming behaviour for *B*. *improvisus* cyprids released in flume flow. Finally, we tested which properties of flow and the length of settling window that best predicted temporary attachment of cypris larvae. We also discuss the possible role of active behaviour to attain attachment.

## Materials and Methods

### Turbulent flow structures in the boundary layer

Near-bed flow in turbulent boundary layers was studied in a recirculating flume (7 m long, 0.5 m wide; see description in Jonsson & Johansson [39]) designed to produce a fully developed turbulent boundary layer at the working section situated 5 m from the entrance. The flume was filled with seawater (salinity 33 ± 1‰, 18 ± 0.5°C) to a depth of 10 cm and turbulence was triggered by a 5 mm bar fixed at the floor shortly after the flume entrance. The flume flow was characterized by mean and instantaneous flow parameters measured using acoustic doppler velocimetry (ADV), and particle image velocimetry (PIV). ADV (20 Hz, Nortek AS) was used to record approximate free-stream velocity at *z* = 30 mm (*z* is height above bed); the mean velocity at this height is at least 90% of the free-stream velocity [40]. ADV measurements were further used to estimate shear velocity (u_*_) from the correlation between the horizontal and vertical fluctuating velocity components ($\bar{u\prime w\prime }$) in the lower part of the turbulent logarithmic layer (*z* = 10 mm), also known as the constant stress layer, according to Schlichting [41]:

$$u_{*}=\sqrt{-\bar{u\prime w\prime }}$$ (1)

The flow characteristics are summarized in Table 1. Finally, we used ADV to check for possible cross-stream differences in flow speed. Within the cross-section used for measurements (minimum 7 cm from the walls), the mean and SD of flow speed differed less than 5% at all free-stream velocities.

**Table 1:** Characteristics of flume flow.

*z* = 30 mm		*z* = 10 mm		
*U*_*∞*_ (m s^-1^)	SD	$\bar{u}$ (m s^-1^)	SD	u_*_ (m s^-1^)
0.05	0.006	0.045	0.007	0.0021
0.10	0.010	0.093	0.011	0.0047
0.15	0.016	0.136	0.016	0.0064
0.20	0.020	0.178	0.020	0.0083

Stream-wise mean velocities ($\bar{u}$) and standard deviations (SD) are given for 2 heights (*z*) above the flume floor. Measurements 30 mm above the flume floor represent approximate free stream velocities (*U*_*∞*_). Data were collected using ADV. Shear velocity (u_*_) is between 4–5% of the free stream velocity.

A main objective was to characterize the instantaneous flow structures at the height of cypris larvae interacting with the bed. This required high-frequency flow measurements very close to the bed (*z* ~ 0.5 mm) of sufficient duration to allow statistical analysis of flow variability. Characterization of turbulent flow structures was performed at free-stream velocities (*U*_*∞*_) of 0.05, 0.1, 0.15 and 0.2 m s^-1^ (± 0.005 m s^-1^) over a smooth flume floor. Velocities at the innermost 5 mm above the bed were measured with PIV; recordings were made in two replicate series of 2 minutes for each free-stream velocity. The water was seeded with 3-μm TiO_2_ tracing particles and the flow was illuminated from above with a double pulsed Nd:YAG laser (Litron, 30 mJ at 532 nm). The beam was expanded by a combination of lenses into a 1 mm thick vertical light sheet situated 7 cm from the transparent sidewall of the flume. The PIV-camera (LaVision Imager Pro X, 1600×1200 pixels) with a Nikon 50-mm Nikkor lens, an extension tube and a 532 nm bandpass filter was run in the double frame mode. Recordings covering an area of 20.8×15.6 mm were done at 14.77 Hz (maximum for the laser at the time) and images were analysed with the DaVis 7.2 software (LaVision) using cross correlation. Correlation calculations were made on a 5×5 mm area at the bed using the multipass option with 50% overlapping interrogation windows. The part closest to the boundary was analysed using the interrogation window size 32×32 pixels followed by 16×16 pixels and for the upper part 64×64 followed by 32×32 was used when necessary for correct vector calculations. To increase the accuracy close to the boundary and because the flow close to the boundary is forced in the horizontal direction, horizontal, elliptical 4:1 Gaussian weighing function interrogation windows were applied. The window size of 16×16 pixels together with the 50% overlap resulted in a resolution of 10 vectors per mm close to the bed. For calculations of instantaneous flow velocities in the stream-wise (*u*) and vertical (*w*) direction at the height of a settling cyprid (*z* = 0.5 mm), an average of 10 vectors (corresponding to a width of 1 mm) was taken from each frame. Integral time scales (based on autocorrelation analysis) for the local flow at *z* = 0.5 mm were 2.1, 0.73, 0.43 and 0.33 s for *U*_*∞*_ of 0.05, 0.1, 0.15 and 0.2 m s^-1^, respectively. Average boundary layer profiles at each free-stream velocity were calculated from the whole 5×5 mm area and the linear part of the boundary layer was determined by eye.

For the resulting velocity series (2 min each, 14.77 Hz) representing stream-wise and vertical velocity fluctuations 0.5 mm above the bed, we quantified the proportion of time occupied by lulls exceeding some critical time interval for increasing tolerable local stress [26]. Crimaldi et al. [26] defined lulls in terms of the time below some critical Reynolds stress which was used as a proxy for the total hydrodynamic forces a settling larva could tolerate to attain successful attachment. Since larvae in all free-stream velocities attached within the linear part of the boundary layer (see Results), we applied the total instantaneous bed shear stress for settlement prediction used by Reidenbach et al. [27]:

$$\tau =\mu \frac{\partial u}{\partial z}-\rho u\prime w\prime$$ (2)

where *τ* is the total bed shear stress (sum of viscous and turbulent stress components), *μ* is the dynamic viscosity of seawater, *∂u/∂z* is the linear gradient in the flow between *u* at *z* = 0.5 mm and the flume floor where *u* = 0 cm s^-1^, and −*ρu'w'* represents the Reynolds stress where *ρ* is the density of seawater and *u'* and *w'* are the instantaneous fluctuations of velocity in the stream-wise and vertical directions, respectively. Total bed shear stress or Reynolds stress may be a suitable proxy for hydrodynamic forcing when the settling mechanism is unknown. In the present paper, however, we also test a specific hypothesis about the settling mechanism involving rheotactic swimming to reduce the relative speed with the substrate. Consequently, besides bed shear stress, we chose to assess instantaneous local velocity as the critical flow property limiting temporary attachment of cypris larvae.

**Fig 2:**
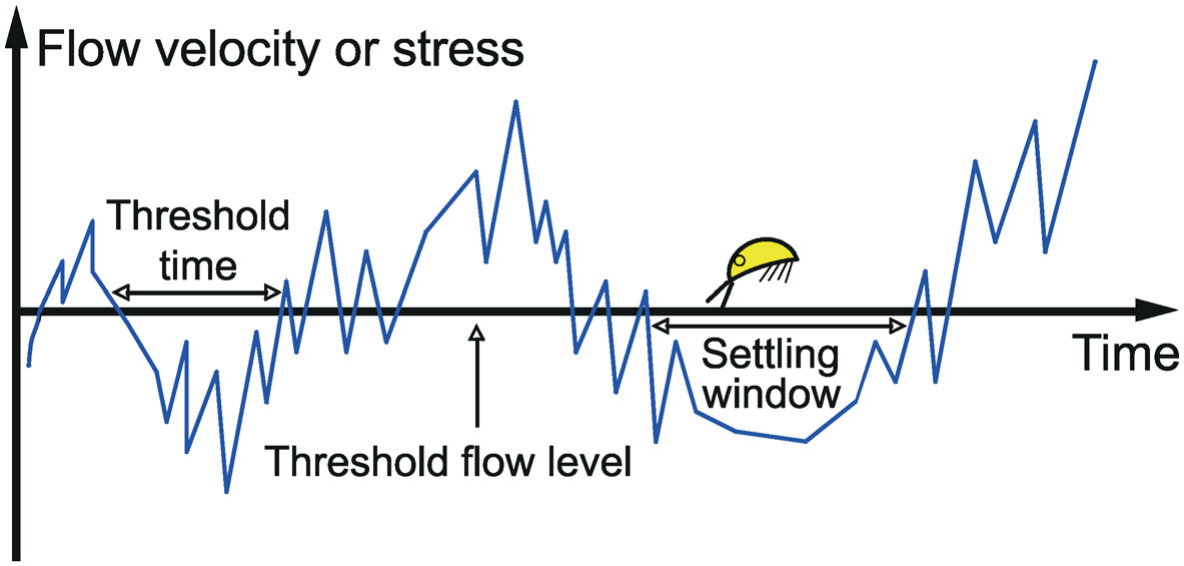
Schematic drawing of the interaction between flow velocity fluctuations and the opportunities for larval attachment. A “stress lull” or “settling window” is defined as the minimum time interval below some critical flow velocity required for successful attachment. According to this hypothesis the cyprid can only use the proportion of time in settling windows for attachment.

The critical time interval is defined as the minimal time a larva needs (*t*_*cr*_), below a certain local velocity or stress, for successful attachment to the substrate (Fig 2). Each time interval below this critical local velocity or stress, which is equal to or longer than *t*_*cr*_ is a settling window (*t*_*w*_). Since the available time during a critical time interval to start and complete the settling event is only *t*_*w*_-*t*_*cr*_, the proportion of time *L(U*_*∞*_,*t*_*cr*_*)* for some free-stream velocity (*U*_*∞*_) available for attachment during a total time interval *T* for a given *t*_*cr*_ is:

$$L\left(U_{\infty },\;t_{cr}\right)=\frac{\sum_{i=1}^{N}\;t_{w_{i}}-Nt_{cr}}{T}$$ (3)

where *N* is the total number of settling windows and *t*_*w*_ > *t*_*cr*_. We examined the proportion of time available for attachment for critical time intervals of 0.14, 0.47, 1.02 and 2.03 s (recordings were made at 14.77 Hz). The two replicate data series for each of the four free-stream velocities showed very similar results and data are given as mean values.

Measurements of local velocities 0.5 mm above the bed were also used to calculate the hydrodynamic forces encountered by cypris larvae attaching to the substrate. The aim was to compare flow-induced forces with previously recorded data of attachment strength in cyprids. For force calculations the cyprids were assumed having a fixed position relative to the substrate (attached) and drag and lift forces were calculated as:

$$F_{D}=0.5C_{D}\rho A_{F}U^{2}$$ (4)

$$F_{L}=0.5C_{L}\rho A_{p}U^{2}$$ (5)

where *F*_*D*_ = drag force, *C*_*D*_ = drag coefficient, *ρ* = fluid density, *A*_*F*_ = frontal area, *U* = fluid velocity experienced by the organism, *F*_*L*_ = lift force, *C*_*L*_ = lift coefficient and *A*_*P*_ = planform area. The frontal and planform areas of the cyprid were calculated from shape and dimensions given in Larsson et al. [42] and for *A*_*F*_ the cyprid was assumed oriented facing the flow. The resulting *A*_*F*_ and *A*_*P*_ from those calculations were 0.05 mm^2^ and 0.1 mm^2^, respectively. Since the Reynolds number (*Re*) for a settling cyprid was well below 1000 (1–100 range) we used the equation for the drag coefficient of a sphere from White [43]:

$$C_{D}=\frac{24}{Re}+\frac{6}{1+Re^{0.5}}+0.4$$ (6)

Following Reidenbach et al. [27] who calculated the hydrodynamic forces on settling nudibranch larvae, a constant *C*_*L*_ value of 0.2 was used. This value of *C*_*L*_ was based on reanalysis of force data [44] on sediment grains sitting on surfaces from Chepil [45].

### Studies of larval adhesion and swimming

Predictions of the probability of attachment from the analysis of turbulent fluctuations in the near-bed flow were tested by studying barnacle cyprids in flume flow. Cyprids of *Balanus improvisus* were reared in the laboratory according to Berntsson et al. [46]. The larvae were aged by storing them in 14°C for 3–5 days before experiments were performed. The working section was fitted with a 50 cm wide and 40 cm long transparent Plexiglas panel covered on the underside with a thin white, opaque plastic coating that was illuminated from below. The purpose of the illumination was to attract the larvae to the flume floor and to facilitate the detection of attached larvae. Cyprids were added to the bed immediately upstream of the working section c. 15 cm from the wall through a glass pipette with an exit diameter of 1 mm. The larvae were added at the flume floor with the pipette pointing horizontally and downstream at 45° with the mainstream direction, which resulted in minimum flow interactions around the pipette exit. Between 20 and 40 larvae were added to the floor during about half a minute. The tip of the pipette was filmed to count the number of added cyprids. The number of larvae found on the 40 cm long illuminated flume floor (working section) 1 min after each release was recorded. The studies were performed at *U*_*∞*_ of 0.05, 0.1, 0.15 and 0.2 m s^-1^ (± 0.005 m s^-1^). Two different batches of cyprids were used and for each batch four experiments at each velocity were performed in random order. In total 210–250 larvae were added per flow speed tested. At *U*_*∞*_ = 0.05 m s^-1^ the local velocity at the bed was so low that the larvae were only slowly drifting in the near-bed flow and could reside in the working section for 1 min without attaching with the antennules. The proportion of larvae found at the bed (93%) thus represented all but those deliberately leaving the working section. Since our hypothesis only includes larvae that attached with their antennules, the larval data at *U*_*∞*_ = 0.05 m s^-1^ were excluded from further analysis. At *U*_*∞*_ of 0.1 m s^-1^ or more, unattached larvae were transported with the near-bed flow. Most larvae reacted by swimming against the flow trying to attach and those not succeeding were swept off the working section with no chance of returning. Proportions of attached cyprids were tested in a 2-factor ANOVA with free-stream velocity as a fixed factor and batch as a random factor. In the analysis a type 1 error (*α*) rate of 0.05 was used. Data were tested for homogeneity of variances using Cochran’s C test and no transformation was needed. A post-hoc test was performed using Student-Newman-Keuls (SNK) procedure.

The swimming speed of cyprids was estimated by releasing larvae in the water column in the flume 5 cm above the illuminated flume floor section. The flume was operating at a low speed since this seemed to trigger swimming towards the attractive illuminated floor better than still water. When larvae started to swim towards the bottom, we used a stopwatch to measure the time it took to reach it. Only larvae that started swimming very soon after release and swam persistently without pausing were included. Our impression was that larvae were very motivated and swam vigorously to reach the bottom. The swimming speed was measured for 16 larvae from 2 different larval batches. The sinking speed of *Balanus improvisus* cyprids is low (0.0013 m s^-1^, unpublished) and is only marginally affecting swimming speed measurements performed in the vertical mode.

The importance of the observed rheotactic swimming response for temporary attachment was quantified in a separate study. Cyprids were added to the bed using the technique with the glass pipette described above. In a free-stream velocity of 0.15 m s^-1^, we observed the larval behaviour immediately preceding successful temporary attachment. A short distance downstream from where larvae were released, a 17 mm section of the flume floor was video-recorded through the flume wall. Larvae were released in 3 series and a total of 29 cyprids attached to the recorded section of the floor. The behaviour preceding temporary attachment was divided into 2 categories; 1) swimming against the current, 2) swimming/drifting downstream or any other behaviour. The result was analysed with a single-variable frequency test (chi-square).

### Comparison between flow analysis of settling windows and empirical larval attachment data

When comparing the predicted proportions of settling windows from the flow analysis with empirical data of larval attachment, the attachment proportions were either used directly or normalized to flow speed. Larval attachment was measured over 40 cm at all free-stream velocities whereas the probability distributions of settling windows are time based. With increasing speed, the time period available for larvae to attach at the working section before being swept off was probably shorter, although this effect was partly reduced by larvae swimming against the flow. To set an upper bound to this bias we converted the length where attachment was possible to a time period possible for attachment by dividing the length of the attachment plate by the average local flow speed at z = 0.5 at the three free-stream velocities. The measured proportions of cyprid attachment were then multiplied by the ratio of the time available for attachment at the free-stream velocity of 0.1 m s^-1^ and the time available at the free-stream velocity the attachment data was recorded for. Hence the attachment data for 0.1 m s^-1^ remained unchanged whereas the attachment data for 0.15 and 0.20 m s^-1^ were increased, compensating for the shorter time available for attachment.

To find the best fit between the empirically determined proportion of larval attachment and the predicted opportunities for attachment from suitable instantaneous flow events, the following procedure was applied. We expected that the attachment probability (*A*_*E*_) is a function of the proportional time available for attachment during lull periods (*L(U*_*∞*_,*t*_*cr*_*)* in Eq 3) with some proportionality constant (*ß*) representing the attachment propensity independent of *L* according to:

$$A_{E}=\beta \cdot L\left(U_{\infty },t_{cr}\right)$$ (7)

Since we have no information on the value of *ß*, we used the relative change in successful attachment of cypris larvae in the different free stream velocities for comparison with predicted opportunities from calculated flow events. The relative changes in attachment of cyprids among free-stream velocities were compared to the relative changes in attachment opportunities among free-stream velocities predicted by the flow events. The ratios between the observed attachment proportion (*A*_*O*_) at *U*_*∞*_ = 0.15 and 0.10 m s^-1^, *U*_*∞*_ = 0.20 and 0.10 m s^-1^, and *U*_*∞*_ = 0.20 and 0.15 m s^-1^ were calculated. From the probability distributions of proportional time available in lull periods (proportional to *A*_*E*_), we calculated similar ratios between the free-stream velocity treatments for each potential critical velocity (0.001–0.1 m s^-1^) or critical shear stress (0.001–0.3 Pa), and for each of the critical time intervals for settlement (0.14, 0.47, 1.02 and 2.03 s). Note that attachment is only expected in a time interval longer than the critical time and below the critical stress or velocity. The critical velocity (or stress) where the *A*_*E*_-based ratio best agreed with the *A*_*O*_-based ratio was noted. Thus 3 critical velocities or stresses were obtained (one for each ratio tested) for each critical time interval. The best fit between the empirically measured attachment and the predicted opportunities from flow events was considered to be the critical time interval where the variation (*SD*) among the 3 critical velocity or stress values was lowest since a low variation indicates that a similar critical velocity or stress was predicted for all three free-stream velocities.

We also wanted to assess if critical velocity or critical shear stress best explained our observed attachment data. Since velocity and stress have different units and this will affect the magnitude of the variation and make a direct comparison difficult, the data were *Z*-transformed:

$$Z=\frac{X_{i}-\bar{X}}{SD}$$ (8)

where *X*_*i*_ is each individual value of critical velocity or stress and $\bar{X}$ and *SD* are the average critical velocity or stress and standard deviation respectively across all critical time intervals. The standard deviations for the *Z*-transformed critical velocities and stresses were then used for the comparison of best fit.

## Results

### Near-bed flow characteristics

**Fig 3:**
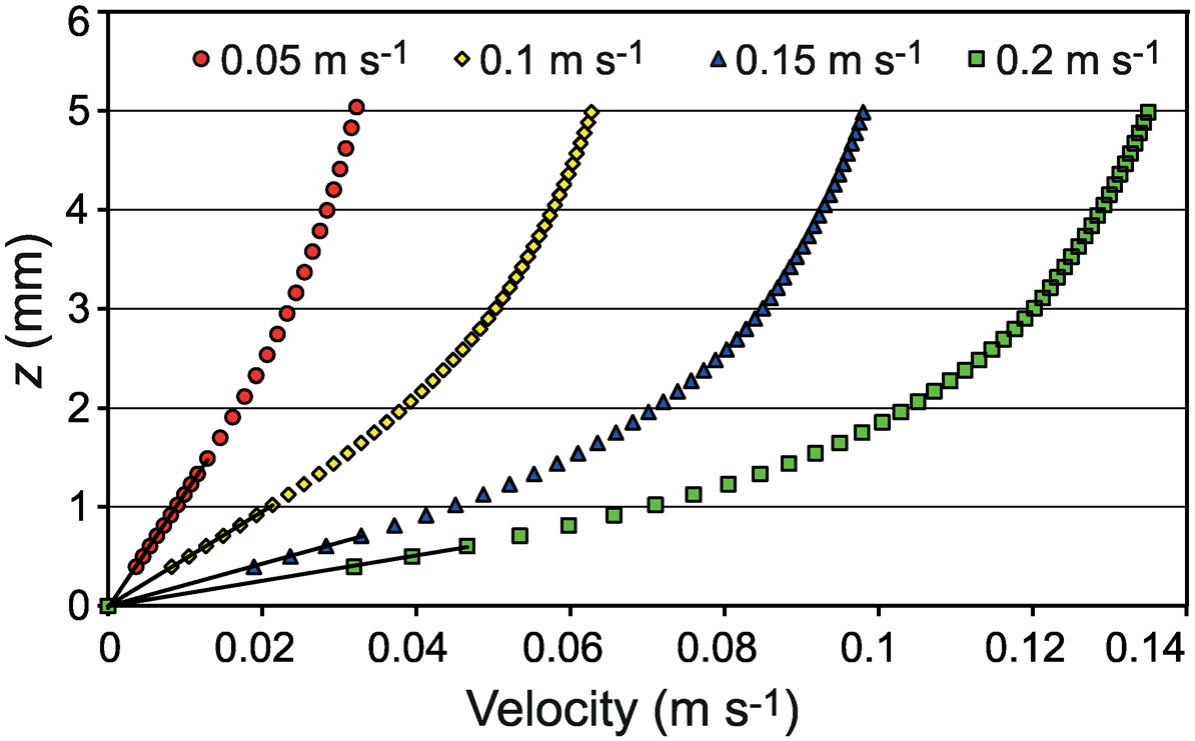
Mean flow velocities in the near-bed region measured by Particle Image Velocimetry (PIV). Velocity gradients are shown for free-stream velocities (*U*_*∞*_) of 0.05, 0.1, 0.15 and 0.2 m s^-1^. The height of the linear part of each velocity profile is indicated by a fitted straight line.

**Fig 4:**
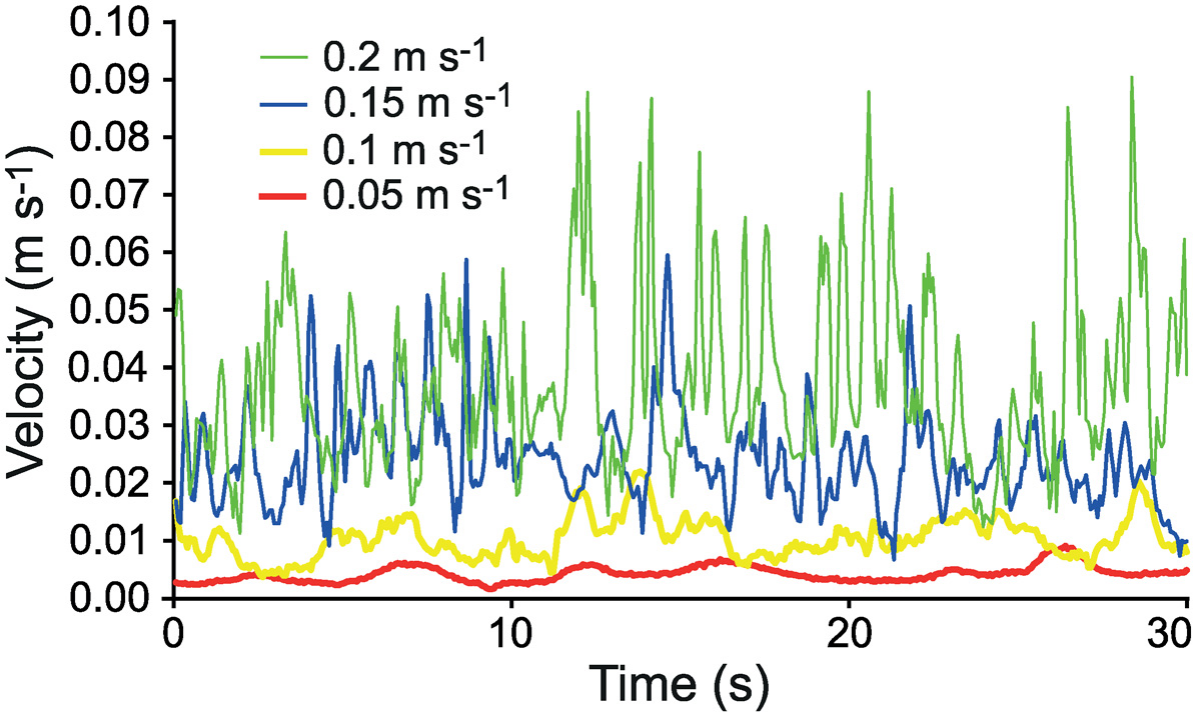
Stream-wise velocity fluctuations at *z* = 0.5 mm above the flume floor. Velocity fluctuations were measured at 15 Hz in free stream velocities (*U*_*∞*_) of 0.05–0.2 m s^-1^ using PIV.

The average flow velocity (from PIV analysis) in the inner part of the boundary layer could be separated into a logarithmic layer and a linear layer close to the bed (Fig 3). Due to the high resolution of the measurements (10 data points mm^-1^), the height of the linear part could be thoroughly determined. The height of the linear part decreased with increasing free-stream velocity and was about 1.6 mm at *U*_*∞*_ = 5 cm s^-1^, 1.1 mm at *U*_*∞*_ = 10 cm s^-1^, 0.8 mm at *U*_*∞*_ = 15 cm s^-1^, and 0.6 mm at *U*_*∞*_ = 20 cm s^-1^. The dimensionless height *z*^+^ (z u_*_/*v*; *v* is kinematic viscosity) was between 3.2 and 4.9, which agrees well with a typical *z*^+^ of about 5 [47]. The results suggest that the cyprids (0.5 mm long) making contact with the bed were within the linear part of the layer at all studied free-stream velocities. Note that although the average velocity increases linearly with height from the substratum where larvae reside at the bed, the velocity fluctuations can be substantial (Fig 4).

### Flume studies of larval attachment success and swimming

**Fig 5:**
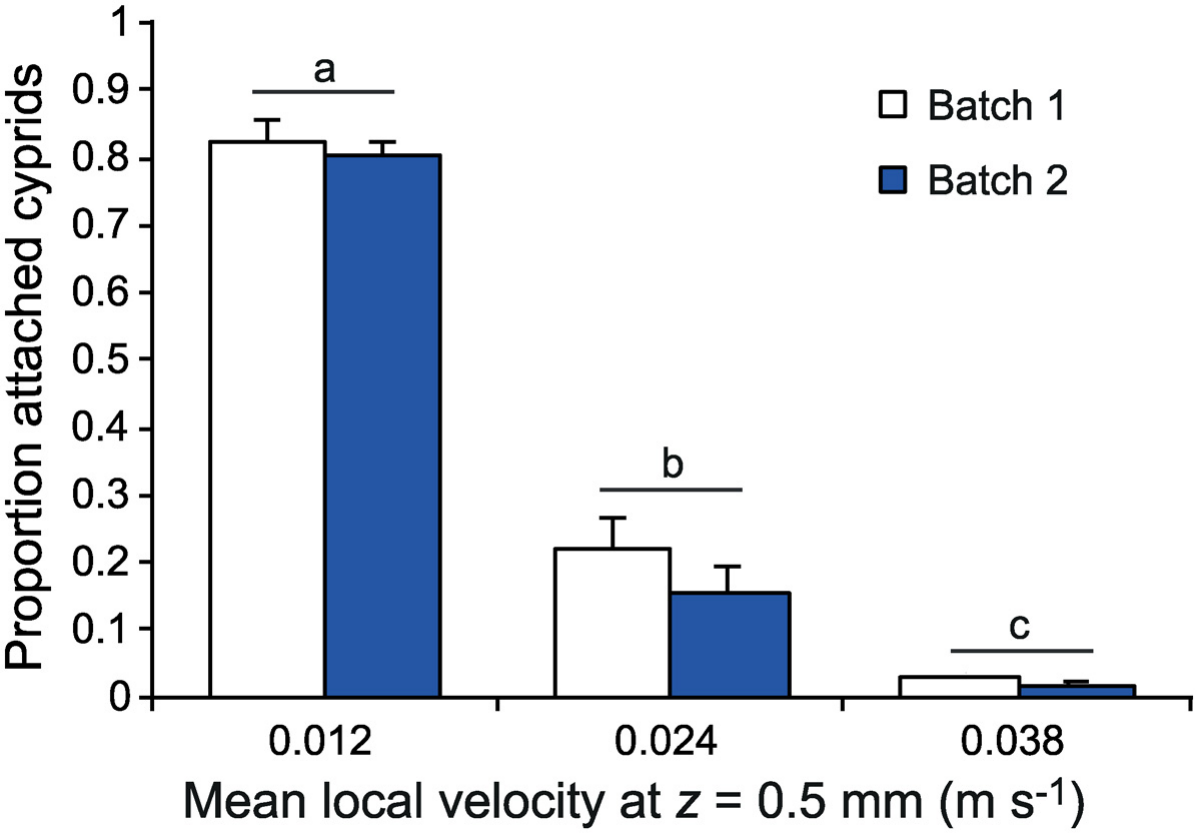
Flow-dependent proportion of temporary attachment in cyprids. Bars represent the proportion (mean ± SE, *n* = 4) of attachment for 20–40 cyprids released in a flume flow at free-stream velocities of 0.1, 0.15 and 0.2 m s^-1^. Lowercase letters denote significant differences among factor levels (*P* < 0.05, SNK post-hoc). The labels show the mean local velocity at the height where cyprids interact with the bed (*z* = 0.5 mm). Data shown are original attachment proportions not normalized to flow speed (see Materials and Methods) and include two larval batches.

Observations of larvae showed that they swam actively against the current and towards the bottom to remain in contact with the bed. We conclude that larvae at *U*_*∞*_ = 0.1 and 0.15 m s^-1^ probably were in contact with the flume floor most of the time whereas at 0.2 m s^-1^ they may have been temporarily resuspended from the bed. When attempting to attach, the studied larvae kept both antennules extended in front of their body. The temporary attachment of cyprids released at the bed decreased sharply with increasing local velocity at *z* = 0.5 mm (2-factor ANOVA, *F*_2,2_ = 1032, *P* = 0.001; Fig 5). The difference was significant among all velocities (SNK post-hoc, *P* 0.05). There was no effect of larval batch and no interaction between larval batch and velocity. The swimming speed of cyprids was estimated to 0.018 ± 0.004 m s^-1^ (mean ± SD). In the detailed study of larval attachment behaviour, swimming against the current was of significant importance to attachment success (*χ*^2^ = 21.6, df = 1, *P* < 0.001). Of the 29 cyprids recorded while temporary attaching to the flume floor, 27 (93%) swam against the current immediately preceding attachment. The other 2 larvae managed to attach while overturning head first putting the antennular discs on the floor downstream of their body.

### Velocity and stress fluctuations and predicted opportunities for larval attachment

**Fig 6:**
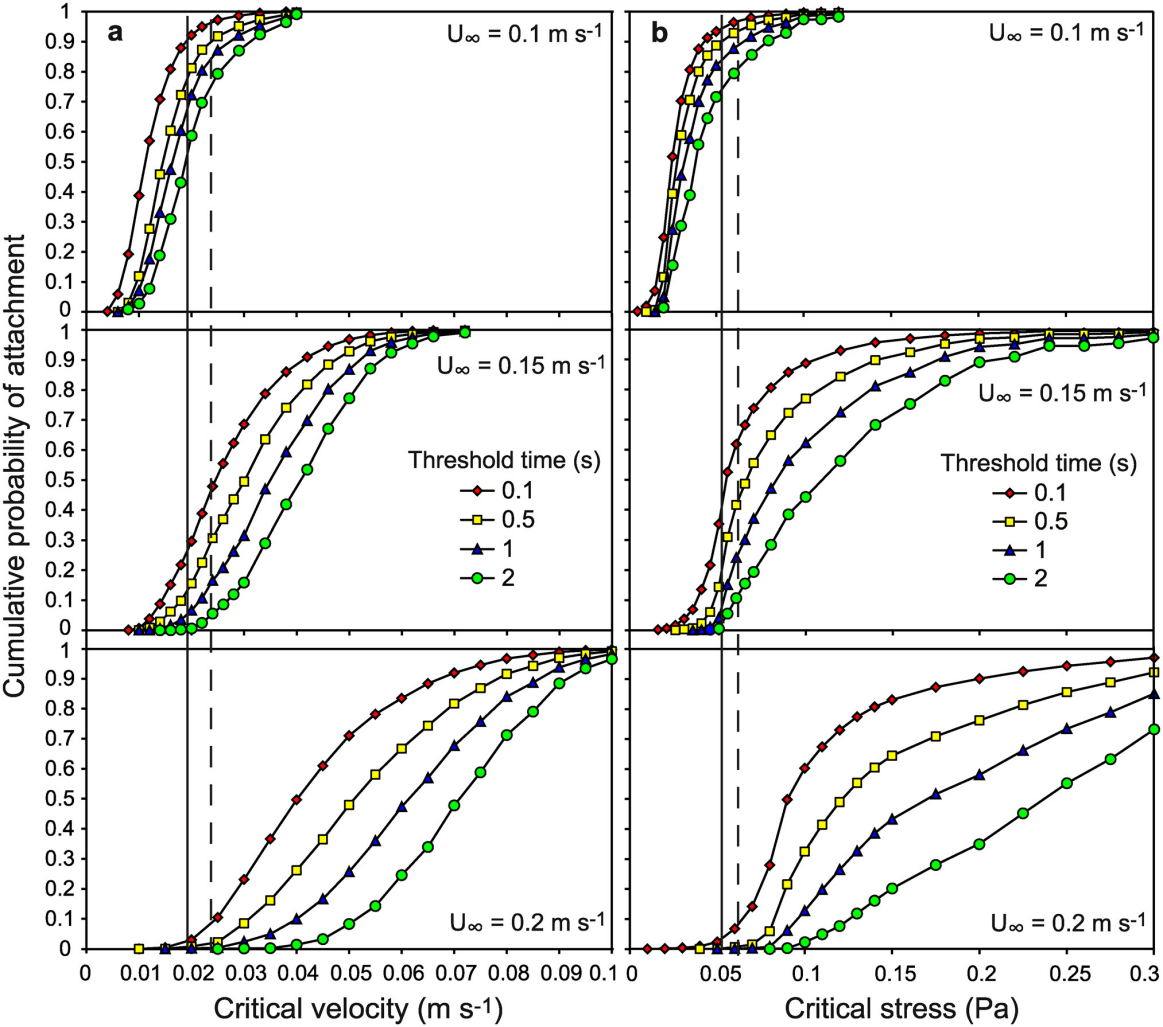
Cumulative probabilities for time in settlement windows. Probabilities displayed as a function of a) tolerable threshold local velocity or b) bed shear stress and minimum time required for temporary attachment. The vertical lines represent the best fit between empirical settlement proportions to the cumulative probability curves for critical velocity and critical stress respectively. The solid lines show best fit based on original attachment data and the dashed lines represent the best fit using attachment proportions normalised to flow speed. See text for more details.

Following the analytical procedure in Crimaldi et al. [26] we produced cumulative frequency distributions for a range of critical instantaneous local velocities (Fig 6a) and instantaneous bed shear stresses (Fig 6b) for various critical time periods required for attachment. These attachment probability distributions were compared to empirical data of cyprid attachment in three free-stream velocities. We used the minimum of standard deviation (on *Z*-transformed data) for the three critical velocities or stresses derived for each critical time interval to find the best fit between the empirical frequencies of larval attachment and the predicted opportunities for attachment from suitable instantaneous flow events. The SD generally decreases with decreasing threshold time needed for attachment indicating cyprids can utilize very short stress lulls (~0.1 s) for attachment (Table 2). The empirical larval attachment data fits better to the distributions of instantaneous velocities than to the distributions of instantaneous bed shear stresses indicating that instantaneous local velocity is the flow property better predicting temporary attachment of cypris larvae compared to total bed shear stresses (Table 2). The best fit (minimum SD) between the original larval attachment data and the frequency of suitable flow events is found for a threshold local flow velocity of 0.019 ± 0.001 m s^-1^ (± SE) for a minimum duration of 0.14 s. If the comparison instead is done using data of temporary attachment normalised to flow speed (see Materials and Methods), a local flow velocity of 0.024 ± 0.000 m s^-1^ for 0.14 s results in the best fit. The vertical lines in the panels of Fig 6 indicate the threshold local flow velocities (a) and the threshold bed shear stresses (b) that best fitted the cyprid attachment data.

**Table 2:** Fit of larval attachment data to critical flow properties.

Attachment data	Critical probability distribution		Threshold attachment time (s)			
			0.14	0.47	1.02	2.03
Original	Velocity	Mean	0.019	0.025	0.031	0.042
		*Z* SD	**0.17**	0.28	0.53	0.95
	Bed shear stress	Mean	0.053	0.069	0.079	0.096
		*Z* SD	0.39	0.70	0.83	1.07
Normalised	Velocity	Mean	0.024	0.030	0.038	0.048
		*Z* SD	**0.054**	0.14	0.47	0.79
	Bed shear stress	Mean	0.062	0.076	0.090	0.12
		*Z* SD	0.37	0.54	0.49	0.63

Average critical velocities (m s^-1^) and bed shear stresses (Pa) derived from comparison between empirically determined larval attachment and predicted opportunities for attachment from suitable instantaneous flow events. The SD-values are from *Z*-transformed data to facilitate comparison of the magnitude of variation between the different units. The best fit (minimum SD) is given in bold for original attachment data and attachment data normalised to flow speed respectively. See text for more details.

### Hydrodynamic forces on larvae

We calculated the resulting hydrodynamic force (vector sum of drag and lift) on attached larvae at 0.024 m s^-1^, which was the most likely threshold velocity for larval attachment (normalised data) and at 0.116 m s^-1^, which was the highest instantaneous flow velocity at *z* = 0.5 mm obtained during our measurements (at *U*_*∞*_ = 20 cm s^-1^). The resulting force at an instantaneous velocity of 0.024 m s^-1^ was 9.3×10^−8^ N and at 0.116 m s^-1^ 7.7×10^−7^ N. To compare these results with cyprid attachment strength we used data produced by Eckman et al. [33] for *Balanus amphiterite* cyprids, which are of equal size as *B*. *improvisus* cyprids. The mean instantaneous force resulting in cyprid dislodgement was 6.0×10^−6^ N. Hence the force imposed on *B*. *improvisus* cyprids in this study at the threshold velocity allowing attachment is two orders of magnitude lower than the average force needed to dislodge temporary attached cyprids in the study by Eckman et al. [33]. The force imposed by the highest instantaneous local velocity measured at *U*_*∞*_ = 0.2 m s^-1^, is still one order of magnitude lower than the force required for dislodgement.

## Discussion

It still remains a major challenge to resolve the relative importance of larval supply, contact rate with the substrate and active habitat choice in determining settlement patterns. Contact with the substrate will strongly depend on coastal circulation, turbulent mixing and gravity. Moreover, behavioural responses may modify contact rate through responses in the water column [17–20] and possibly also close to the substrate [37]. A crucial step is to after initial contact maintain a position at the substrate long enough to ensure a more secure temporary or permanent attachment [3]. The least understood step is how the transition between initial contact and a secure attachment is acquired and how it is affected by flow regime, surface properties and larval behaviour.

In the present study we found a decline in temporary attachment of barnacle larvae at mean free-stream flow velocities between 0.1 to 0.2 m s^-1^. The gradual decline without a sudden threshold suggests that cypris larvae either show large individual variability in their ability to make an attachment or that they respond to non-mean flow properties. Crimaldi et al. [26] suggested that larval attachment in a turbulent boundary layer depends on the presence of sufficiently long lull events below some critical stress. Thus, the frequency of time in these settling windows in a given flow regime should determine the probability of successful attachment. The distribution of suitable instantaneous flow events is strongly affected by unidirectional flow speed, wave exposure and presence of microhabitats [26–28]. The instantaneous flow structures in Crimaldi et al. [26] were measured in the turbulent logarithmic part of the boundary layer, but in accordance with Reidenbach et al. [27], our measurements clearly illustrate that also within the linear part of the boundary layer, where larval attachment usually takes place, flow velocities in the stream-wise direction vary substantially (Fig 4). We conclude from the fit to observed larval attachment probabilities in Fig 6 that a fixed response to instantaneous flow velocity, in this case lulls below 0.019–0.024 m s^-1^, can explain the gradual decline in attachment probability with increasing flow speeds. Furthermore we can conclude that instantaneous velocity is a better predictor of cyprid attachment probability compared to instantaneous bed shear stress (Table 2). This could also be true for other larvae that are strong swimmers whereas instantaneous stress may be more important for larvae incapable of rheotaxis. We also calculated the drag and lift forces on larvae and found that the resulting hydrodynamic force imposed on cyprids at the critical local flow velocity allowing attachment is two orders of magnitudes lower than the hydrodynamic force needed to dislodge cyprids during temporary attachment [33]. This indicates that attachment strength is not an important factor limiting settlement of cyprids in turbulent flow. Therefore models of settlement probability based on measurements of larval attachment strength, e.g. [27], may overestimate settlement probability in cases where the crucial limiting step precedes temporary or permanent attachment.

Instead a limiting step can be the transition between initial contact and temporary (or permanent) attachment and this study illuminates the importance of investigating near-surface larval behaviour and larval attachment mechanisms when predicting larval settling probabilities from flow properties. For barnacle cyprids, we here show that this transition is likely governed by rheotactic swimming allowing the cyprid to remain in contact with the substrate until a stress lull can be utilized for temporary attachment. In this study we repeatedly observed how cyprids of *B*. *improvisus* swam upstream when close to the bed, a behaviour that was found very important for accomplishing temporary attachment (>90% of observed attachments were immediately preceded by upstream swimming). Interestingly, the predicted critical local flow velocity allowing temporary attachment is close to the swimming speed, 0.018 m s^-1^, of *B*. *improvisus* cyprids. Although larvae swam vigorously in our swimming speed measurements, the measured speed is probably somewhat lower than the absolute maximum larvae can generate when encountering very challenging conditions. Crisp also observed that cyprids of the barnacle *Semibalanus balanoides* showed strong negative rheotaxis [37]. Crisp suggested that cyprids in this way may adjust their swimming speed to the ambient flow to facilitate settlement [48]. Our results support this hypothesis and based on our findings we propose a scenario where the cyprid upon contact with the substrate gains a reference to exercise negative rheotactic swimming. This reduces the relative speed to the substrate and while orienting the body to a suitable position the cyprid can make a strong temporary attachment with its antennular discs. This attachment is likely facilitated by the highly flexible joints between segments of the antennules [49]. According to our analysis the whole sequence can be completed in only 0.1 s allowing settlement also in challenging flow environments. The maximum swimming velocity that cyprids may orient in an upstream direction in response to substrate contact may thus determine at what flow speed barnacles can settle. In a downwelling flume, DiBacco et al. [50] found that the upward swimming velocity of *S*. *balanoides* increased with velocity of the downwelling flow and could be as high as 0.072 m s^-1^ for short time periods. Crisp [37] measured swimming speeds for *S*. *balanoides* and *Balanus crenatus* that were more than twice as fast as the speeds we measured for *B*. *improvisus*. An additional aspect is that larger larvae will experience faster local flow speeds because of the boundary layer. Cyprids of *S*. *balanoides* and *B*. *crenatus* are about twice the size of *B*. *improvisus* cyprids so in a linear velocity gradient their swimming speeds should allow settlement at similar free-stream velocities. Crisp [37] also measured the probability of temporary attachment but in laminar pipe-flow. The flow regime in the narrow glass tubes used in Crisp [37] was very different from the flow in our study, but from the velocity gradient shown in his Fig 4 the local flow speed, where temporary attachment for *S*. *balanoides* cyprids declined, should be close to his measured swimming speed of 0.05 m s^-1^.

Many rocky-bottom intertidal barnacle species inhabit natural environments where ambient flow velocities due to wave action or tidal currents often greatly exceed the flow velocities investigated in the present study. With increasing flow velocity, the frequency of lull periods in the near bed flow with speeds lower than the cyprid swimming speed decreases and eventually becomes almost nonexistent. Hence our results suggest that cyprids in order to attach in high-flow environments might rely on the periods when waves and tides are turning. It may further be speculated that fast swimming bursts against the boundary-layer flow is an adaptation to allow cyprids to actively select relatively high-flow environments. It also seems likely that cyprids have the ability to fine-tune their flow exposure during temporary attachment by searching for optimum sites among surface roughness elements [51], or by actively leaving a substrate exposed to excessive flow speeds; cyprids settling in high flow speeds may suffer reduced feeding rates as post-metamorphic juveniles [24]. Few marine larvae rival barnacle cyprids in terms of swimming speed. Ascidian tadpole larvae may reach above 0.01 m s^-1^ [52] and could exploit a similar settlement behaviour as cyprids, although no information is yet available on a possible rheotactic behaviour. Also species with slow-swimming larvae can colonize high-flow environments, e.g. hydroids and bryozoa [53]. Little is known about the mechanisms governing the transition between initial contact and secure attachment in most larvae but it can be speculated that hydrophobic interactions, mucous strings and ciliated body surfaces are involved [35,54] and subsequently many larvae can strengthen the attachment by adhesive glands, e.g. in bivalves and in bryozoa [55,56]. However, in contrast to barnacle cyprids, larvae adopting a strategy based on high surface adhesion will not have the same flexibility to select habitats based on flow speed.

In conclusion, we here for the first time compare settlement predictions from flow analysis with actual attachment of larvae in turbulent flow. Our study suggests that marine larvae less than one mm in size can exercise behavioural responses at the bed that enhance attachment probability. Directed swimming against the flow allows barnacle cyprids to utilize very short periods of velocity lulls in the turbulent flow for attachment. The relative importance of larval delivery rate to the substratum and larval behavioural responses before and after temporary attachment on settlement patterns remains challenging to estimate. However, although high turbulence levels increase the rate of larval transport to the substrate, Crimaldi et al. [26] found that the decrease in attachment probability due to high turbulence had a larger effect on settlement success than the increase in larval transport caused by turbulent mixing. Hence the ability of barnacle larvae to increase the attachment rate by behavioural actions at the substrate raises the possibility to control habitat choice and can modify relationships between larval supply and recruitment.

## Supporting Information

S1 Dataset Underlying data. The raw data underlying the statistical analyses, tables and figures in the present study.
(XLSX)
